# Supplementary material for: Deletion of wfs1 Impairs Oligodendrocyte Precursor Cells Dorsal Distribution and Myelination Through the wfs1-hmgcs1 Axis in Zebrafish
Source: Biology (Basel). 2026 Aug 16;15(16):1402. doi: 10.3390/biology15161402 (PMC13510151; doi:10.3390/biology15161402)
Supplement: Supplementary file 1 [file biology-15-01402-s001.zip › File S1-Transcriptome sequencing data and differentially expressed genes (DEGs) in wfs1-- zebrafish at 5 dpf/2.KEGG/wfs1KOvsWT/GSEA/WT/VEGF_SIGNALING_PATHWAY/VEGF_SIGNALING_PATHWAY(DRE04370).html]

Details for gene set VEGF\_SIGNALING\_PATHWAY(DRE04370)[GSEA]

|  || Dataset | deg.sample |
| Phenotype | sample.cls |
| Upregulated in class | WT |
| GeneSet | VEGF\_SIGNALING\_PATHWAY(DRE04370) |
| Enrichment Score (ES) | -0.25563586 |
| Normalized Enrichment Score (NES) | -1.1412692 |
| Nominal p-value | 0.21030928 |
| FDR q-value | 0.5343695 |
| FWER p-Value | 1.0 |
Table: GSEA Results Summary

  

Fig 1: Enrichment plot: VEGF\_SIGNALING\_PATHWAY(DRE04370)      
 Profile of the Running ES Score & Positions of GeneSet Members on the Rank Ordered List

  

| PROBE | DESCRIPTION (from dataset) | GENE SYMBOL | GENE\_TITLE | RANK IN GENE LIST | RANK METRIC SCORE | RUNNING ES | CORE ENRICHMENT || 1 | 556226\_1 | cdc42l2 |  |  | 680 | 2.623 | 0.0146 | No |
| 2 | 100006675 | prkcba |  |  | 711 | 2.563 | 0.0489 | No |
| 3 | 100000252\_3 | pik3r3b |  |  | 716 | 2.550 | 0.0838 | No |
| 4 | 793158\_1 | cdc42l |  |  | 826 | 2.351 | 0.1126 | No |
| 5 | 562552 | zgc:171775 |  |  | 839 | 2.330 | 0.1442 | No |
| 6 | 559020 | ptgs2b |  |  | 1021 | 2.074 | 0.1670 | No |
| 7 | 100000252 | pik3r3b |  |  | 1965 | 1.382 | 0.1564 | No |
| 8 | 798864\_1 | pla2g4f.1 |  |  | 2559 | 1.155 | 0.1536 | No |
| 9 | 246227 | ptgs2a |  |  | 2612 | 1.141 | 0.1676 | No |
| 10 | 565241 | ppp3ccb |  |  | 3535 | 0.922 | 0.1512 | No |
| 11 | 447814 | ppp3r1b |  |  | 3939 | 0.841 | 0.1501 | No |
| 12 | 100333254 | nfatc2a |  |  | 4008 | 0.829 | 0.1594 | No |
| 13 | 404211 | pik3r2 |  |  | 4059 | 0.817 | 0.1690 | No |
| 14 | 793648 | LOC793648 |  |  | 4799 | 0.705 | 0.1555 | No |
| 15 | 559087 | pla2g4ab |  |  | 4877 | 0.697 | 0.1626 | No |
| 16 | 562011\_1 | shc2 |  |  | 4943 | 0.688 | 0.1700 | No |
| 17 | 565386\_1 | pla2g4f.2 |  |  | 5440 | 0.638 | 0.1632 | No |
| 18 | 100148581 | LOC100148581 |  |  | 5502 | 0.634 | 0.1699 | No |
| 19 | 564921 | bada |  |  | 5627 | 0.623 | 0.1746 | No |
| 20 | 561747 | plcg2 |  |  | 5761 | 0.610 | 0.1788 | No |
| 21 | 58100 | badb |  |  | 5892 | 0.598 | 0.1829 | No |
| 22 | 100002318 | mapk13 |  |  | 6155 | 0.572 | 0.1825 | No |
| 23 | 565241\_1 | ppp3ccb |  |  | 6607 | 0.527 | 0.1756 | No |
| 24 | 30682 | vegfaa |  |  | 6618 | 0.526 | 0.1825 | No |
| 25 | 325084 | src |  |  | 6875 | 0.502 | 0.1813 | No |
| 26 | 30554 | pla2g4aa |  |  | 7169 | 0.476 | 0.1787 | No |
| 27 | 557926\_2 | ppp3cca |  |  | 7193 | 0.474 | 0.1844 | No |
| 28 | 561737 | pik3ca |  |  | 7195 | 0.474 | 0.1909 | No |
| 29 | 327321 | mapkapk2a |  |  | 7206 | 0.473 | 0.1971 | No |
| 30 | 798864 | pla2g4f.1 |  |  | 7791 | 0.438 | 0.1847 | No |
| 31 | 562552\_1 | zgc:171775 |  |  | 7885 | 0.432 | 0.1877 | No |
| 32 | 100006132 | si:ch211-1f22.2 |  |  | 8076 | 0.417 | 0.1875 | No |
| 33 | 557926 | ppp3cca |  |  | 8563 | 0.398 | 0.1777 | No |
| 34 | 399546 | pxna |  |  | 9208 | 0.353 | 0.1623 | No |
| 35 | 378972 | akt2 |  |  | 9293 | 0.347 | 0.1644 | No |
| 36 | 557648 | mapkapk2b |  |  | 9880 | 0.311 | 0.1502 | No |
| 37 | 65237\_1 | mapk14a |  |  | 10301 | 0.285 | 0.1409 | No |
| 38 | 554230 | kdr |  |  | 10614 | 0.265 | 0.1347 | No |
| 39 | 393228\_1 | zgc:55558 |  |  | 10754 | 0.257 | 0.1339 | No |
| 40 | 563181 | map2k2a |  |  | 10792 | 0.256 | 0.1362 | No |
| 41 | 497384 | prkca |  |  | 10900 | 0.249 | 0.1363 | No |
| 42 | 393953 | prkcbb |  |  | 11901 | 0.196 | 0.1075 | No |
| 43 | 100003514 | prkcg |  |  | 12176 | 0.181 | 0.1014 | No |
| 44 | 30681 | mapk12a |  |  | 13899 | 0.097 | 0.0485 | No |
| 45 | 100149794 | akt3b |  |  | 14249 | 0.080 | 0.0387 | No |
| 46 | 266756 | mapk14b |  |  | 14849 | 0.053 | 0.0205 | No |
| 47 | 100331840 | sphk1 |  |  | 15103 | 0.041 | 0.0131 | No |
| 48 | 30380 | nras |  |  | 15635 | 0.015 | -0.0033 | No |
| 49 | 373867 | plcg1 |  |  | 16057 | -0.004 | -0.0165 | No |
| 50 | 791141 | zgc:158659 |  |  | 16140 | -0.006 | -0.0190 | No |
| 51 | 565386 | pla2g4f.2 |  |  | 16232 | -0.009 | -0.0218 | No |
| 52 | 558154 | vegfab |  |  | 16935 | -0.032 | -0.0434 | No |
| 53 | 795167 | rac3b |  |  | 17171 | -0.040 | -0.0503 | No |
| 54 | 368243 | hspb1 |  |  | 17509 | -0.054 | -0.0601 | No |
| 55 | 394174 | pik3cd |  |  | 17974 | -0.074 | -0.0737 | No |
| 56 | 142986 | ptk2ab |  |  | 18232 | -0.087 | -0.0806 | No |
| 57 | 327321\_1 | mapkapk2a |  |  | 18364 | -0.092 | -0.0835 | No |
| 58 | 404211\_1 | pik3r2 |  |  | 18555 | -0.101 | -0.0881 | No |
| 59 | 415185 | mapk11 |  |  | 18679 | -0.107 | -0.0904 | No |
| 60 | 327204 | rac1a |  |  | 19053 | -0.124 | -0.1005 | No |
| 61 | 560549 | akt3a |  |  | 19324 | -0.136 | -0.1071 | No |
| 62 | 399480 | mapk3 |  |  | 19514 | -0.144 | -0.1111 | No |
| 63 | 406728 | map2k1 |  |  | 19540 | -0.145 | -0.1099 | No |
| 64 | 571865 | LOC571865 |  |  | 19757 | -0.155 | -0.1145 | No |
| 65 | 557109 | raf1b |  |  | 20203 | -0.175 | -0.1261 | No |
| 66 | 336839 | cdc42 |  |  | 20515 | -0.189 | -0.1333 | No |
| 67 | 794574 | ppp3ca |  |  | 20620 | -0.195 | -0.1339 | No |
| 68 | 492789 | zgc:101699 |  |  | 20960 | -0.213 | -0.1416 | No |
| 69 | 550554 | ppp3cb |  |  | 21144 | -0.223 | -0.1443 | No |
| 70 | 100150090 | sphk2 |  |  | 22316 | -0.285 | -0.1773 | No |
| 71 | 360144\_1 | mapk1 |  |  | 23446 | -0.357 | -0.2079 | No |
| 72 | 100002263 | pik3cb |  |  | 23508 | -0.361 | -0.2048 | No |
| 73 | 557176 | pik3r1 |  |  | 23610 | -0.367 | -0.2030 | No |
| 74 | 65237 | mapk14a |  |  | 23905 | -0.386 | -0.2069 | No |
| 75 | 790934 | mapkapk3 |  |  | 24314 | -0.415 | -0.2140 | No |
| 76 | 100002225 | map2k2b |  |  | 24736 | -0.433 | -0.2213 | No |
| 77 | 492763 | casp9 |  |  | 25122 | -0.455 | -0.2272 | No |
| 78 | 557810 | mapk12b |  |  | 25647 | -0.498 | -0.2369 | No |
| 79 | 30716 | raf1a |  |  | 25882 | -0.518 | -0.2371 | No |
| 80 | 550286 | hrasa |  |  | 26472 | -0.575 | -0.2477 | Yes |
| 81 | 100333967 | si:ch73-374l24.1 |  |  | 26564 | -0.584 | -0.2426 | Yes |
| 82 | 437027 | rac3a |  |  | 26579 | -0.586 | -0.2350 | Yes |
| 83 | 399546\_1 | pxna |  |  | 26961 | -0.626 | -0.2384 | Yes |
| 84 | 386705 | ptk2aa |  |  | 26978 | -0.627 | -0.2302 | Yes |
| 85 | 110439839 | LOC110439839 |  |  | 27117 | -0.641 | -0.2258 | Yes |
| 86 | 415151 | rac2 |  |  | 27490 | -0.672 | -0.2283 | Yes |
| 87 | 553656 | hrasb |  |  | 27554 | -0.680 | -0.2209 | Yes |
| 88 | 497384\_1 | prkca |  |  | 27854 | -0.723 | -0.2204 | Yes |
| 89 | 562838 | rac1b |  |  | 28146 | -0.763 | -0.2191 | Yes |
| 90 | 556226 | cdc42l2 |  |  | 28186 | -0.770 | -0.2097 | Yes |
| 91 | 101910198 | akt1 |  |  | 28441 | -0.810 | -0.2066 | Yes |
| 92 | 100006208 | LOC100006208 |  |  | 28738 | -0.866 | -0.2040 | Yes |
| 93 | 565130 | pxnb |  |  | 28810 | -0.880 | -0.1942 | Yes |
| 94 | 100006166 | si:ch211-1f22.1 |  |  | 28986 | -0.917 | -0.1871 | Yes |
| 95 | 445289 | kras |  |  | 29269 | -0.994 | -0.1823 | Yes |
| 96 | 403146 | akt2l |  |  | 29342 | -1.012 | -0.1707 | Yes |
| 97 | 564337 | ppp3r1a |  |  | 29604 | -1.097 | -0.1638 | Yes |
| 98 | 792699 | rac1l |  |  | 30004 | -1.247 | -0.1592 | Yes |
| 99 | 100006675\_1 | prkcba |  |  | 30078 | -1.276 | -0.1440 | Yes |
| 100 | 100148818 | si:ch73-55i23.1 |  |  | 30770 | -1.736 | -0.1419 | Yes |
| 101 | 58100\_1 | badb |  |  | 31292 | -2.420 | -0.1251 | Yes |
| 102 | 100000252\_1 | pik3r3b |  |  | 31434 | -2.733 | -0.0920 | Yes |
| 103 | 565241\_2 | ppp3ccb |  |  | 31577 | -3.181 | -0.0528 | Yes |
| 104 | 793158 | cdc42l |  |  | 31794 | -4.556 | 0.0030 | Yes |
Table: GSEA details [plain text format]

  

Fig 2: VEGF\_SIGNALING\_PATHWAY(DRE04370)      
 Blue-Pink O' Gram in the Space of the Analyzed GeneSet

  

Fig 3: VEGF\_SIGNALING\_PATHWAY(DRE04370): Random ES distribution      
 Gene set null distribution of ES for **VEGF\_SIGNALING\_PATHWAY(DRE04370)**
